# Factors influencing health-related quality of life after gastrectomy for cancer

**DOI:** 10.1007/s10120-017-0771-0

**Published:** 2017-10-24

**Authors:** Hylke J. F. Brenkman, Juul J. W. Tegels, Jelle P. Ruurda, Misha D. P. Luyer, Ewout A. Kouwenhoven, Werner A. Draaisma, Donald L. van der Peet, Bas P. L. Wijnhoven, Jan H. M. B. Stoot, Richard van Hillegersberg, on behalf of the LOGICA Study Group

**Affiliations:** 1 Department of Surgery, Division Cancer Center, University Medical Center Utrecht, Heidelberglaan 100, 3508 GA Utrecht, The Netherlands; 2 Department of Surgery, Zuyderland Medical Center, Sittard, The Netherlands; 3 Department of Surgery, Catharina Hospital, Eindhoven, The Netherlands; 4 Department of Surgery, Ziekenhuisgroep Twente, Almelo, The Netherlands; 5 Department of Surgery, Meander Medical Center, Amersfoort, The Netherlands; 6 Department of Surgery, VU Medical Center, Amsterdam, The Netherlands; 7 Department of Surgery, Erasmus Medical Center, Rotterdam, The Netherlands

**Keywords:** Gastric cancer, Gastrectomy, Quality of life

## Abstract

**Aim:**

Insight in health-related quality of life (HRQoL) may improve clinical decision making and inform patients about the long-term effects of gastrectomy. This study aimed to evaluate and identify factors associated with HRQoL after gastrectomy.

**Methods:**

This cross-sectional study used prospective databases from seven Dutch centers (2001–2015) including patients who underwent gastrectomy for cancer. Between July 2015 and November 2016, European Organization for Research and Treatment of Cancer HRQoL questionnaires QLQ-C30 and QLQ-STO22 were sent to all surviving patients without recurrence. The QLQ-C30 scores were compared to a Dutch reference population using a one-sample *t* test. Spearman’s rank test was used to correlate time after surgery to HRQoL, and multivariable linear regression was performed to identify factors associated with HRQoL.

**Results:**

A total of 222 of 274 (81.0%) patients completed the questionnaires. Median follow-up was 29 months (range, 3–171) and 86.9% of patients had a follow-up >1 year. The majority of patients had undergone neoadjuvant treatment (64.4%) and total gastrectomy (52.7%). Minimally invasive gastrectomy (MIG) was performed in 50% of the patients. Compared to the general population, gastrectomy patients scored significantly worse on most functional and symptom scales (*p* < 0.001) and slightly worse on global HRQoL (78 vs. 74, *p* = 0.012). Time elapsed since surgery did not correlate with global HRQoL (Spearman’s ρ = 0.06, *p* = 0.384). Distal gastrectomy, neoadjuvant treatment, and MIG were associated with better HRQoL (*p* < 0.050).

**Conclusion:**

After gastrectomy, patients encounter functional impairments and symptoms, but experience only a slightly impaired global HRQoL. Distal gastrectomy, the ability to receive neoadjuvant treatment, and MIG may be associated with HRQoL benefits.

**Electronic supplementary material:**

The online version of this article (doi:10.1007/s10120-017-0771-0) contains supplementary material, which is available to authorized users.

## Introduction

Gastric cancer is the fifth most common type of cancer worldwide [1], and surgical resection is the cornerstone of treatment with curative intent. Because survival after gastrectomy for cancer has improved since the introduction of a more extensive lymphadenectomy and perioperative chemotherapy [2, 3], the quality of life following treatment has become increasingly important. Various complaints that are associated with the treatment, such as a loss of appetite, early satiety, reflux, dysphagia, nausea, and change of stools, may have a profound impact on patient health-related quality of life (HRQoL) [4].

HRQoL is multidimensional, comprising physical, medical, psychological, and social parameters secondary to a disease and its treatment, and is considered as one of the most important outcome measurements in cancer treatment nowadays. Evaluating HRQoL helps health professionals to focus on certain aspects to improve current treatment strategies. Moreover, understanding HRQoL in patients after surgery for gastric cancer is useful for informing patients about the (long-term) risks and benefits of an intervention.

High-quality studies evaluating HRQoL after gastrectomy for cancer in Western countries are scarce. In addition, it is unknown if the HRQoL of patients after gastrectomy differs from HRQoL in the general population. The aim of this multicenter cross-sectional study was therefore to evaluate HRQoL in patients who underwent gastrectomy for cancer in relationship to the general population and to identify factors influencing HRQoL.

## Methods

### Study design

This cross-sectional multicenter cohort study included data from seven referral centers for gastric cancer surgery in the Netherlands. All data were extracted from prospectively collected institutional databases. Institutional Review Board approval was obtained, and informed consent requirement was waived for this study.

### Patient population

This study included patients who underwent gastrectomy for gastric adenocarcinoma between 2001 and 2015. Only patients who were alive and free of disease were approached for participation. According to national guidelines, patients received either perioperative treatment and gastrectomy or gastrectomy alone [5]. Staging was performed in accordance with the 7th American Joint Committee on Cancer TNM staging system [6, 7]. Information on patient, treatment, and histopathological characteristics were collected from the prospective institutional databases. Follow-up was updated retrospectively.

### Quality of life

Cross-sectional HRQoL was measured by means of the European Organisation for Research and Treatment of Cancer (EORTC) questionnaires. The EORTC-QLQ-C30 was developed to assess the HRQoL of patients with cancer in general [8]; the EORTC-QLQ-STO22 was developed to assess the HRQoL of patients with gastric cancer specifically [4]. The EORTC-QLQ-C30 and EORTC-QLQ-STO22 consist of 30 and 22 questions, respectively, scored on a 4- or 7-point Likert scale, which are translated to a global QoL scale, functional scales (physical, role, emotional, cognitive, social, and body image), and symptom scales (fatigue, nausea and vomiting, pain, dyspnea, insomnia, appetite loss, constipation, diarrhea, financial difficulties, dysphagia, reflux, eating restrictions, anxiety, dry mouth, taste, and hair loss). Higher scores in global HRQoL and functional scales indicate better HRQoL, whereas higher scores in the symptom scales indicate more severe symptoms. Patients were contacted to complete the questionnaires by mail, and received one reminder by telephone in the case of no response.

### Statistical analyses

HRQoL data obtained by the questionnaires were scored according to the manual [9]. The patients’ HRQoL scores of the EORTC-QLQ-C30 were compared to a general Dutch reference population consisting of 1731 individuals by performing one-sample *t* tests [10]. Subgroup analyses were performed on age and gender, and of patients who had a follow-up ≥12 months. Based on previous studies, a difference in HRQoL >10 points was considered clinically relevant [11]. A Spearman’s rank correlation coefficient (ρ) was calculated to measure the dependence of different HRQoL scores to time since surgery. A positive or negative correlation coefficient was considered very strong if ρ > 0.8, strong if ρ = 0.6–0.8, moderate if ρ = 0.4–0.6, weak if ρ = 0.2–0.4, and very weak if ρ < 0.2. Finally, multivariable linear regression analysis with stepwise backward elimination was performed to evaluate the association between HRQoL scales and patient, surgical, and histopathological variables. These variables were chosen based on their possible association with HRQoL as demonstrated by previous studies [12–16]. Major complications were defined as complications requiring a re-intervention or intensive care admission. A *p* value < 0.05 was considered statistically significant. All statistical analyses were performed using IBM SPSS version 21.

## Results

### Patients

A total of 683 patients who underwent gastrectomy for gastric adenocarcinoma were identified from the combined database. Some 409 patients were excluded because of death or recurrent disease. The remaining 274 patients were invited to participate in the study and were sent HRQoL questionnaires. The questionnaires were completed by 222 of 274 (81.0%) patients (Supplementary file 1).

### Baseline

**Fig. 1:**
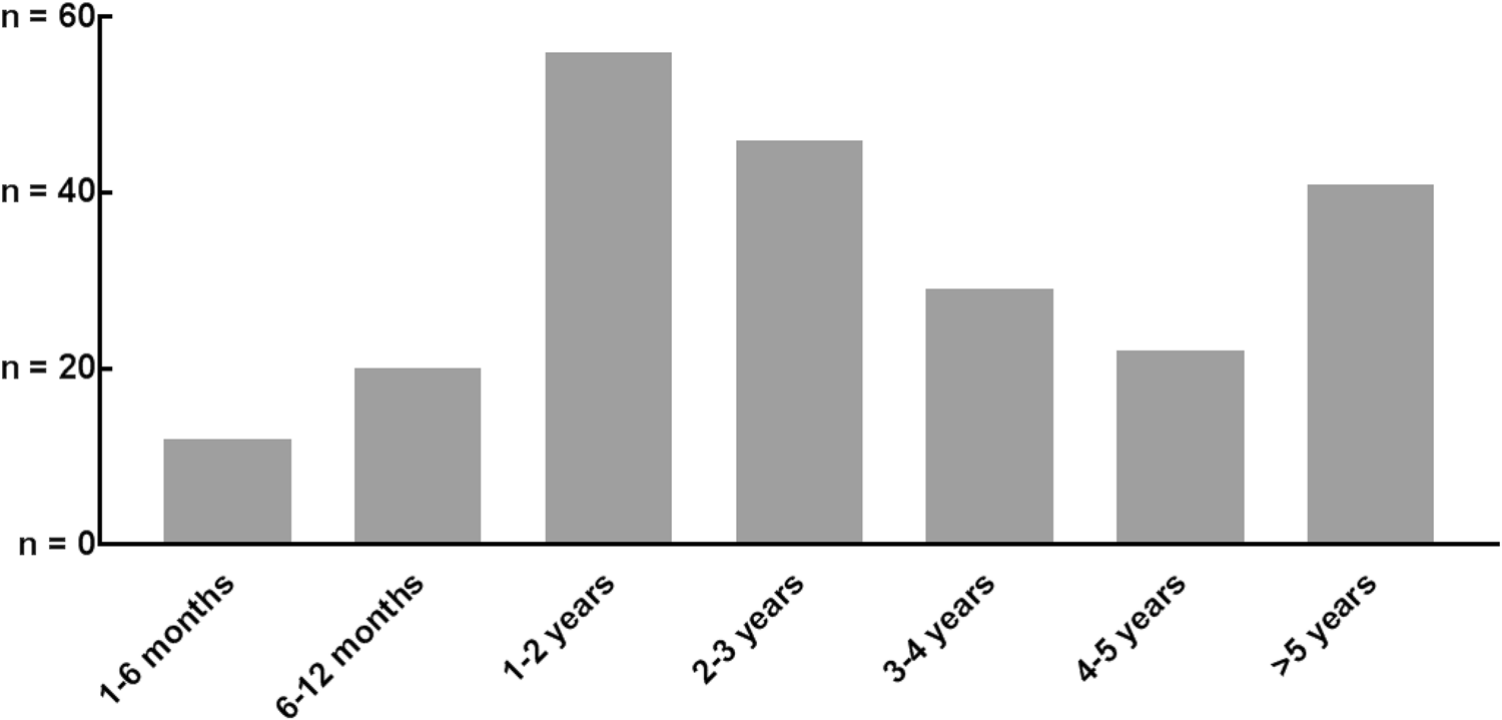
Timing of quality of life (QoL) questionnaire (months after surgery)

There were no differences in patient and tumor characteristics, nor in postoperative outcomes, of respondents (*n* = 222) and non-respondents (*n* = 52). The median follow-up at completing the questionnaires was 29 months (range, 3–171); 193 (86.9%) of the respondents had a follow-up >1 year, whereas 40 (18.0%) had a follow-up >5 years (Fig. 1). Patient, surgical, and histopathological characteristics of the patients are shown in Table 1. Some 143 (64.4%) patients had undergone neoadjuvant treatment, 117 patients (52.7%) a total gastrectomy, and 111 patients (50.0%) a minimally invasive gastrectomy (MIG). Of the patients receiving perioperative treatment, most received perioperative chemotherapy according to a regimen similar to epirubicin, cisplatin, and capecitabin [3]. Radiotherapy was not routinely performed, except for some patients who received adjuvant chemoradiation as part of the CRITICS trial [17]. The majority of patients (*n* = 119, 53.6%) had an advanced tumor stage (≥II). Major postoperative complications were seen in 42 (18.9%) patients. Median hospital stay was 9 days (range, 3–124), and 24 (10.8%) patients were readmitted within 30 days after discharge.

**Table 1:** Baseline characteristics of 222 patients who underwent gastrectomy for cancer

Factor	*n* = 222 (%)
Age (years) [mean (± SD)]	67.7 (± 10.6)
BMI (kg/m^2^) [mean (± SD)]	25.5 (± 4.4)
Gender	
Male	141 (63.5)
Female	81 (36.5)
Malignancy history	
No	157 (86.3)
Yes	25 (14.7)
Unknown	40
Comorbidity	169 (76.1)
Cardiovascular	110 (49.5)
Pulmonary	37 (16.7)
Diabetes mellitus	37 (16.7)
ASA	
I	33 (14.9)
II	141 (63.5)
III	46 (20.7)
IV	2 (0.9)
Neoadjuvant treatment	143 (64.4)
Adjuvant treatment	67 (30.2)
Year of surgery	
2001–2004	3 (11.4)
2005–2009	10 (4.5)
2010–2012	66 (29.7)
2013–2015	143 (64.4)
Surgical type	
Distal gastrectomy	105 (47.3)
Total gastrectomy	117 (52.7)
Surgical approach	
Open	111 (50.0)
Laparoscopic	111 (50.0)
Complications	89 (40.1)
Major	42 (18.9)
Anastomotic leakage	16 (7.2)
Pulmonary	24 (10.8)
Hospital stay (median, range) (days)	9 (3–124)
Radicality	
R0	214 (96.4)
R+	5 (2.3)
Missing	3 (1.4)
pTNM stage	
0	15 (6.8)
I	88 (39.6)
II	78 (35.1)
III	40 (18.0)
IV	1 (0.5)

### HRQoL versus reference population

**Fig. 2:**
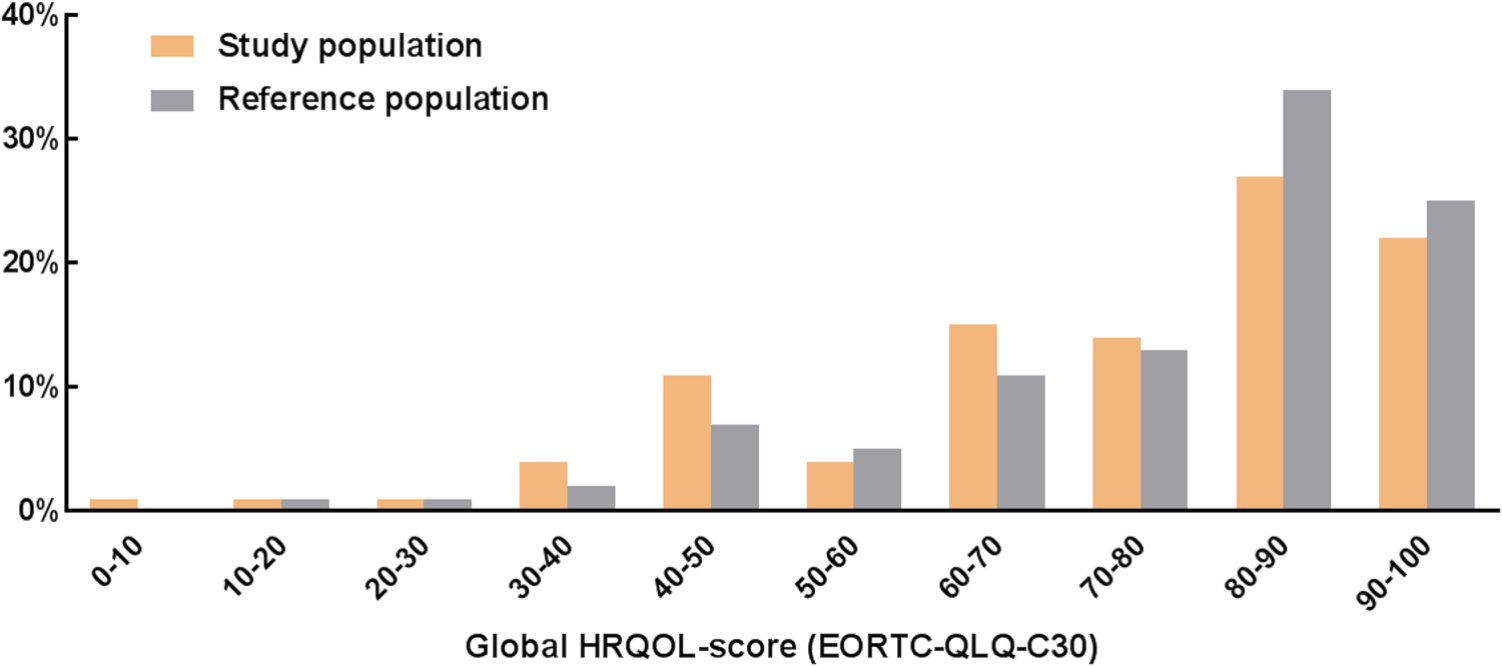
Distribution of global HRQOL-scores (EORTC-QLQ-C30) of 222 patients who underwent gastrectomy for cancer compared to the general Dutch population

The mean HRQoL score and standard deviation of the study population and the reference population are shown in Table 2 [10]. Patients who underwent gastrectomy for cancer had statistically significantly lower scores than the reference population for all functional scales (*p* < 0.001) and most general symptom scales (*p* < 0.002), expect for pain symptoms (*p* = 0.067) (Table 2). This difference was clinically relevant for most scales (physical, role, cognitive, and social functioning; fatigue, nausea, and vomiting, dyspnea, appetite loss, diarrhea, and financial difficulties). On global HRQoL, patients scored significantly worse compared to the reference population, although this difference was clinically irrelevant (weight mean difference = 4, *p* = 0.002). The distribution of global HRQoL scores is shown in Fig. 2: 78% of patients scored a global HRQoL >60, compared to 83% in the reference population (*p* = 0.027).

**Table 2:** Mean (standard deviations) health-related quality of life (HRQoL) scores of 222 patients who underwent gastrectomy for cancer compared to the general Dutch population

	Total *n* = 222	Reference population *n* = 1731	WMD	*p* value
Quality of life questionnaire (QLQ)-C30				
Global quality of life^a^	74 [21]	78 [17]	−4	**0.002**
Functional scales^a^				
Physical	79 [20]	90 [15]	−11	**<0.001**
Role	73 [29]	89 [21]	−16	**<0.001**
Emotional	81 [24]	89 [16]	−8	**<0.001**
Cognitive	81 [22]	92 [15]	−11	**<0.001**
Social	81 [26]	94 [16]	−13	**<0.001**
General symptom scales^b^				
Fatigue	33 [27]	17 [20]	+16	**<0.001**
Nausea and vomiting	14 [22]	2.7 [10]	+11	**<0.001**
Pain	18 [26]	15 [22]	+3	0.067
Dyspnoea	18 [25]	7.1 [17]	+11	**<0.001**
Insomnia	20 [29]	14 [23]	+6	**0.002**
Appetite loss	21 [32]	3.3 [12]	+18	**<0.001**
Constipation	10 [22]	4.8 [14]	+5	**0.001**
Diarrhea	18 [26]	3.9 [14]	+14	**<0.001**
Financial difficulties	16 [29]	3.1 [13]	+13	**<0.001**
QLQ-STO22				
Functional scales^a^				
Body image	82 [28]	NA		NA
General symptom scales^b^				
Dysphagia	16 [21]	NA		NA
Pain	20 [22]	NA		NA
Reflux	22 [25]	NA		NA
Eating restrictions	25 [25]	NA		NA
Anxiety	30 [27]	NA		NA
Dry mouth	21 [30]	NA		NA
Taste	17 [29]	NA		NA
Hair loss	18 [32]	NA		NA

Scores are presented as mean [± SD]

^a^ Score range 0–100: higher scores represent a better quality of life or level of functioning

^b^ Score range 0–100: higher scores represent more severe symptoms

Bold values indicate significant variables (*p* < 0.05)

In a subgroup analysis of men or women compared to a reference population of comparable age (60–69 years), similar results (as demonstrated in Table 2) were found (Supplementary files 2 and 3). In addition, subgroup analyses of patients with a follow-up ≥12 months also did not influence the results (Supplementary file 4).

### HRQoL and time elapsed since surgery

Spearman rank correlation coefficients between HRQoL and time after surgery were calculated, demonstrating weak correlations ranging from −0.13 to +0.13 (Table 3). In patients with follow-up <1 year, a larger variation of correlations was present, ranging from −0.46 to +0.29. Moreover, there was no difference in HRQoL between the years of surgery (*p* = 0.523).

**Table 3:** Spearman’s rank correlation coefficients between quality of life and length of follow-up (FU) since surgery

	Spearman’s rank coefficient (ρ)		
	Total cohort (*n* = 222)	Follow-up <1 year (*n* = 29)	Follow-up >1 year (*n* = 193)
Quality of life questionnaire (QLQ)-C30			
Global quality of life^a^	+ 0.06	− 0.01	+ 0.11
Functional scales^a^			
Physical	− 0.08	− 0.11	− 0.03
Role	− 0.02	− 0.17	+ 0.06
Emotional	+ 0.07	− 0.11	+ 0.14
Cognitive	+ 0.08	+ 0.10	+ **0.20**
Social	+ 0.03	− 0.03	+ 0.07
General symptom scales^b^			
Fatigue	+ 0.08	+ 0.29	− 0.01
Nausea and vomiting	+ 0.07	− 0.14	+ 0.06
Pain	+ 0.06	+ 0.03	− 0.01
Dyspnea	+ 0.03	+ 0.17	− 0.03
Insomnia	+ 0.04	− 0.11	− 0.05
Appetite loss	− 0.02	− 0.25	− 0.05
Constipation	− 0.07	− 0.17	− 0.08
Diarrhea	+ 0.07	− 0.06	+ 0.09
Financial difficulties	− 0.02	− 0.14	− 0.06
QLQ-STO22			
Functional scales^a^			
Body image	+ 0.03	+ 0.25	+ 0.02
General symptom scales^b^			
Dysphagia	− 0.03	− 0.32	− 0.03
Pain	− 0.02	− 0.16	− 0.09
Reflux	+ 0.13	+ 0.01	+ 0.13
Eating restrictions	− 0.11	− 0.11	− 0.07
Anxiety	− 0.11	− 0.03	− 0.13
Dry mouth	+ 0.02	− 0.03	+ 0.03
Taste	− 0.13	+ 0.05	− 0.13
Hair loss	− 0.05	− **0.46**	− 0.03

*FU* follow-up

Bold values indicate significant variables (*p* < 0.05)

### Predictive factors for HRQoL

Global HRQoL was significantly higher in patients who underwent distal gastrectomy [+6.5, 95% CI (0.8;12), *p* = 0.026] and neoadjuvant treatment [+8.2, 95% CI (1.8;15), *p* = 0.012]. Moreover, both distal gastrectomy and neoadjuvant treatment were associated with better HRQoL scores in other symptom and functioning scales (Table 4). Minimally invasive surgery resulted in significantly better HRQoL scores on physical functioning [+7.5 (2.4;12), *p* = 0.004], fatigue [−7.0 (−14;−0.05), *p* = 0.048], pain [−9.1 (−16;−2.6), *p* = 0.007], and dry mouth [−9.5 (−17;−1.8), *p* = 0.015]. Furthermore, female gender and a high ASA score were predictive factors for impaired HRQoL in some scales.

**Table 4:** Multivariable linear regression model on quality of life, symptom scales, and functional scales from the EORTC QLQ-C30 and QLQ-STO2

	Global QoL	Physical functioning	Role functioning	Emotional functioning	Cognitive functioning	Social functioning
A. Quality of life and functional scales from the EORTC QLQ-C30 questionnaire^a^						
Female gender	–	–5.1 [− 10;0.2]	**–**	**–**	–	–
Higher age	+0.3 [− 0.03;0.6]	–	–	**+0.4 [0.1;0.8]**	–	–
Distal gastrectomy	**+6.5 [0.8;12]**	+4.7 [− 0.4;9.8]	**+8.9 [1.1;17]**	–	–	–
Minimally invasive surgery	–	**+7.5 [2.4;12]**	–	–	–	–
Neoadjuvant therapy	**+8.2 [1.8;15]**	–	–	+6.1 [− 1.0;13]	–	–
ASA score	–	**–6.1 [−** **10;-2.0]**	–	–	–	–
Major complication	–	–	–	**+8.2 [0.3;16]**	–	–
Higher tumor stage	–	–	+4.4 [− 0.1;8.8]	–	–	–
>1 year after surgery	–	–	**−** **12 [−** **24;−** **1.0]**	–	–	–

	Fatigue	Nausea and vomiting	Pain	Dyspnea	Insomnia	Appetite loss	Constipation	Diarrhea	Financial problems
B. Symptom scales from the EORTC QLQ-C30 questionnaire^b^									
Female gender	+7.1 [− 0.04;14]	**+7.9 [2.1;14]**	–	–	**+17 [9.2;24]**	**+12 [3.4;20]**	–	**+8.9 [1.8;16]**	–
Higher age	–	**−** **0.5 [−** **0.7;−** **0.2]**	**−** **0.4 [−** **0.8;−** **0.1]**	–	–	–	–	–	**–0.7 [−** **1.1;−** **0.4]**
Distal gastrectomy	**−** **8.7 [−** **16;−** **1.7]**	− 5.0 [− 11;0.8]	–	**+6.8 [0.1;14]**	–	**−** **15 [−** **23;−** **6.9]**	–	**−** **10 [−** **17;−** **3.1]**	–
Minimally invasive surgery	**−** **7.0 [−** **14;−** **0.05]**	–	**−** **9.1 [−** **16;−** **2.6]**	–	–	–	–	–	–
Neoadjuvant therapy	− 6.6 [− 14;0.9]	**−** **11 [−** **18;−** **4.8]**	**−** **11 [−** **19;−** **3.6]**	**−** **7.8 [−** **15;−** **0.8]**	–	–	–	**−** **9.4 [−** **17;−** **2.1]**	–
Higher ASA score	–	–	–	–	–	–	–	–	+5.5 [−0.7–12]
Major complication	–	–	–	–	–	–	–	+7.9 [− 0.8;17]	–
Higher tumor stage	–	–	–	–	–	–	–	–	–
>1 year after surgery	**+11 [0.8;22]**	–	–	–	**+12 [1.3;23]**	–	–	–	–

	Body image^a^	Dysphagia	Pain	Reflux	Eating restrictions	Anxiety	Dry mouth	Taste	Hair loss
C. Body image and symptom scales from the EORTC QLQ-STO22 questionnaire^b^									
Female gender	**−** **8.6 [−** **16;-1.0]**	+5.2 [− 0.4;11]	+4.9 [− 0.9;11]	–	**+11 [4.0;17]**	+7.2 [− 0.1;15]	–	+7.2 [− 0.5;15]	**+23 [15;31]**
Higher age	**+0.5 [0.2;0.9]**	–	–0.3 [− 0.5;0.01]	–	–	**–0.4 [−** **0.7;−** **0.02]**	–	**+0.5 [0.2;0.9]**	–
Distal gastrectomy	–	**−** **12 [−** **17;−** **6.3]**	**−** **7.0 [−** **13;−** **1.1]**	**−** **9.9 [−** **17;−** **3.2]**	**−** **17 [−** **23;−** **10]**	− 6.4 [− 14;0.9]	**–**	**−** **12 [−** **20;−** **4.6]**	–
Minimally invasive surgery	–	–	–	–	–	–	**−** **9.5 [−** **17;−** **1.8]**	–	–
Neoadjuvant treatment	–	–	–	**−** **15 [−** **22;−** **8.4]**	**−** **7.7 [−** **15;−** **0.9]**	–	–	–	+7.7 [− 0.6;16]
ASA score	− 5.7 [− 12;0.5]	–	–	–	–	–	**+9.5 [3.3;16]**	–	–
Major complication	–	–	–	–	–	–	–	–	–
Higher tumor stage	–	–	–	− 3.1 [− 6.8;0.5]	–	–	–	–	–
>1 year after surgery	–	–	+8.2 [− 0.2;17]	+9.2 [− 0.2;19]	–	–	–	–	–

Scores are presented as linear regression coefficients, with 95% confidence intervals between brackets. During stepwise backward linear regression, the weakest associated variables are excluded from the model (–)

^a^ Higher scores represent better quality of life or functioning

^b^ Higher scores represent more symptoms

Bold values indicate significant variables (*p* < 0.05)

## Discussion

This cross-sectional multicenter study demonstrates that patients who underwent gastrectomy experienced functional complaints and symptoms, whereas global HRQoL was only slightly lower compared to the Dutch reference population. Most importantly, distal gastrectomy and the ability to receive neoadjuvant treatment were associated with higher global HRQoL, whereas minimally invasive surgery was associated with better functional and symptom scores.

The results of this study are relevant, as they may represent the average population undergoing gastrectomy for cancer in the West. Unfortunately, to date, most studies on HRQoL after gastrectomy lacked data on patient characteristics [12], were performed in the Asian population [13–15], or selected patients by age [14], minimally invasive procedures [13, 14], comorbidities [13, 14], tumor stage [15], or perioperative chemotherapy [16]. The present study is the first study to include all disease-free patients who underwent gastrectomy for cancer and to compare the results to a general Western reference population.

This study demonstrated that the absolute difference in global HRQoL compared to the general population is clinically irrelevant. These findings correspond with previous studies on HRQoL after other types of cancer surgery, such as esophagectomy [18] and breast cancer surgery [19], and demonstrate that patients can achieve a high life satisfaction after surviving cancer, regardless of the presence of symptoms or functional impairment. This adjustment might be explained by a phenomenon called reconceptualization, which is well known in HRQoL assessments [20]. Reconceptualization implies that certain circumstances, such as surviving cancer, can alter the internal reference points of an individual’s well-being that are critical for an evaluation of HRQoL. As a result, objective impairments in different areas of life can be measured, but the overall HRQoL is based on other factors and can therefore be compared to data before the circumstance or from the general population [14].

HRQoL benefits of minimally invasive gastrectomy over open gastrectomy were also demonstrated by an Asian randomized controlled trial [15]. Comparable benefits were found on physical functioning and symptoms. However, benefits in global HRQoL could not be confirmed by the present study. This difference could be explained because the present study evaluated a population with longer follow-up, whereas one might expect global HRQoL benefits of minimally invasive surgery mainly in the short term. Although minimally invasive gastrectomy may have additional advantages over open gastrectomy [21–25], results from more randomized controlled trials are awaited [26–28].

Other factors possibly influencing HRQoL identified in this study were the extent of gastrectomy, neoadjuvant treatment, gender, and time since surgery. During distal gastrectomy, a functional part of the stomach is preserved and a less extensive lymphadenectomy is required [29], contributing to a higher HRQoL compared to total gastrectomy on several domains [13, 30]. An interesting finding is the higher HRQoL in patients who were able to receive neoadjuvant treatment, compared to patients who did not receive neoadjuvant treatment. Although chemotherapy has been shown to increase HRQoL in the palliative setting [31], the only evidence available in the curative setting demonstrates that physical fitness of patients decreases during neoadjuvant treatment [32]. The lower HRQoL after gastrectomy found in women is consistent with previous studies and the general reference population [10, 18]. Elapsed time since surgery did not correlate with HRQoL, which may be a result of the long-term follow-up of patients in this study. Previous studies have demonstrated that time since surgery is mainly associated with HRQoL in the early recovery period [16], whereas QoL remains relatively stable after the first year following surgery [33].

There are some limitations of this study that need to be addressed. First, although subgroup analyses of males and females of comparable age were performed, we cannot exclude confounding caused by differences between the study population and general population in baseline. Second, because patients with undiagnosed recurrent disease might have been included, this factor could have decreased the HRQoL measured in the study population [16]. Third, despite correcting for confounding factors, selection bias cannot be fully excluded. For instance, selection bias might explain the higher HRQoL in patients who received neoadjuvant treatment. Another form of selection bias might be caused by the inclusion of mainly long-term survivors, which may have led to an overestimation of the quality of life of patients following gastrectomy in this study. Last, the cross-sectional design of this study did not allow the analysis of repeated measurements of HRQoL over time.

In conclusion, although surviving patients experience significant symptoms and functional problems after gastrectomy, global HRQoL is more or less comparable to the general population. Distal gastrectomy, the ability to receive neoadjuvant treatment, and minimally invasive surgery could be associated with HRQoL benefits and may therefore be preferred. The results of this study may help healthcare professionals during clinical decision making and the preoperative process of informed consent.

## Electronic supplementary material

Below is the link to the electronic supplementary material.

Supplementary material 1 (DOCX 61 kb)
