# Supplementary material for: Factors influencing health-related quality of life after gastrectomy for cancer
Source: Gastric Cancer. 2017 Oct 24;21(3):524–32. doi: 10.1007/s10120-017-0771-0 (PMC5906484; doi:10.1007/s10120-017-0771-0)
Supplement: Supplementary file 1 — Supplementary material 1 (DOCX 61 kb) [file 10120_2017_771_MOESM1_ESM.docx]

**Supplementary files**

**Supplementary file 1.** Study flow diagram


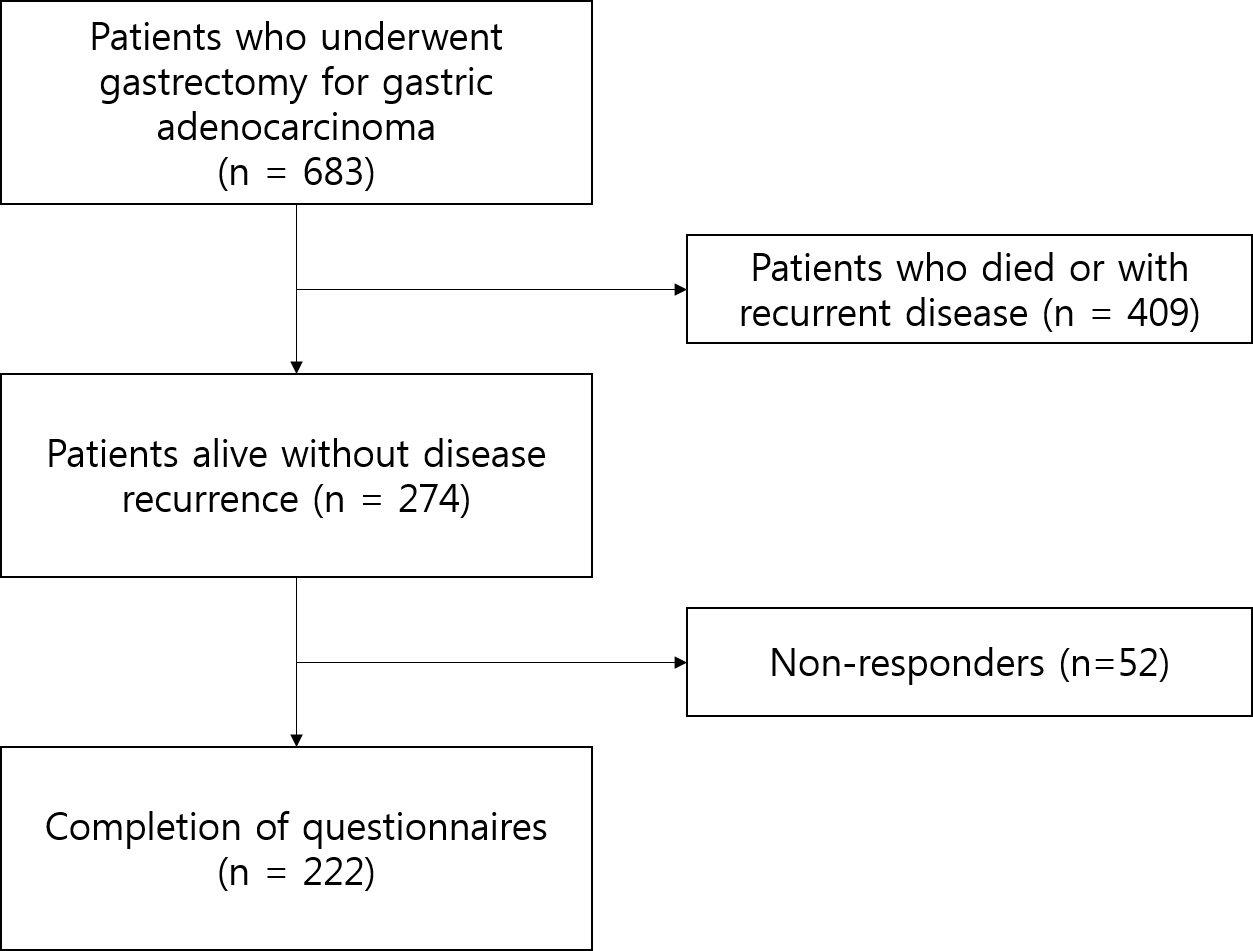


| **Supplementary file 2.** Mean [standard deviations] health-related quality of life (HRQOL)-scores of 141 male patients who underwent gastrectomy for cancer compared to a general Dutch population consisting of males aged 60-69. | | | | | | |
| --- | --- | --- | --- | --- | --- | --- |
|  | **Total**  ***n* = 141** | | **Reference population**  ***n*= 216** | | **WMD** | ***p*-value** |
| **Quality of life questionnaire (QLQ)-C30** |  |  |  |  |  |  |
|  |  |  |  |  |  |  |
| **Global quality of life^a^** | 75 | [20] | 81 |  | – 6 | **0.001** |
|  |  |  |  |  |  |  |
| **Functional scales^a^** |  |  |  |  |  |  |
| Physical | 80 | [20] | 93 |  | – 13 | **<0.001** |
| Role | 76 | [28] | 92 |  | – 16 | **<0.001** |
| Emotional | 82 | [24] | 91 |  | – 9 | **<0.001** |
| Cognitive | 81 | [23] | 92 |  | – 11 | **<0.001** |
| Social | 82 | [26] | 96 |  | – 14 | **<0.001** |
|  |  |  |  |  |  |  |
| **General symptom scales^b^** |  |  |  |  |  |  |
| Fatigue | 31 | [25] | 12 |  | + 19 | **<0.001** |
| Nausea and vomiting | 11 | [20] | 1.5 |  | + 9 | **<0.001** |
| Pain | 16 | [25] | 11 |  | – 5 | **0.011** |
| Dyspnoea | 17 | [22] | 7.1 |  | + 10 | **<0.001** |
| Insomnia | 14 | [25] | 11 |  | + 3 | 0.157 |
| Appetite loss | 17 | [31] | 2.2 |  | + 15 | **<0.001** |
| Constipation | 10 | [23] | 3.4 |  | + 7 | **0.001** |
| Diarrhoea | 15 | [24] | 2.5 |  | + 12 | **<0.001** |
| Financial difficulties | 15 | [30] | 2.9 |  | + 12 | **<0.001** |
|  |  |  |  |  |  |  |
| Scores are presented as mean [±SD]. a) Score range 0-100, higher scores represent a better quality of life or level of functioning; b) Score range 0-100, higher scores represent more severe symptoms. | | | | | | |

| **Supplementary file 3.** Mean [standard deviations] health-related quality of life (HRQOL)-scores of 81 female patients who underwent gastrectomy for cancer compared to a general Dutch population consisting of females aged 60-69. | | | | | | |
| --- | --- | --- | --- | --- | --- | --- |
|  | **Total**  ***n* = 81** | | **Reference population**  ***n*= 153** | | **WMD** | ***p*-value** |
| **Quality of life questionnaire (QLQ)-C30** |  |  |  |  |  |  |
|  |  |  |  |  |  |  |
| **Global quality of life^a^** | 71 | [22] | 77 |  | – 6 | **0.027** |
|  |  |  |  |  |  |  |
| **Functional scales^a^** |  |  |  |  |  |  |
| Physical | 77 | [20] | 85 |  | – 8 | **<0.001** |
| Role | 70 | [32] | 84 |  | – 14 | **<0.001** |
| Emotional | 77 | [24] | 88 |  | – 11 | **<0.001** |
| Cognitive | 82 | [21] | 91 |  | – 9 | **<0.001** |
| Social | 78 | [28] | 93 |  | – 15 | **<0.001** |
|  |  |  |  |  |  |  |
| **General symptom scales^b^** |  |  |  |  |  |  |
| Fatigue | 37 | [29] | 19 |  | + 19 | **<0.001** |
| Nausea and vomiting | 19 | [24] | 2.1 |  | + 17 | **<0.001** |
| Pain | 21 | [27] | 25 |  | – 4 | 0.241 |
| Dyspnoea | 21 | [29] | 7.8 |  | + 13 | **<0.001** |
| Insomnia | 31 | [33] | 20 |  | + 11 | **0.004** |
| Appetite loss | 28 | [34] | 3.9 |  | + 24 | **<0.001** |
| Constipation | 12 | [21] | 8.1 |  | + 4 | 0.150 |
| Diarrhoea | 23 | [30] | 3.7 |  | + 19 | **<0.001** |
| Financial difficulties | 19 | [27] | 2.6 |  | + 16 | **<0.001** |
|  |  |  |  |  |  |  |
| Scores are presented as mean [±SD]. a) Score range 0-100, higher scores represent a better quality of life or level of functioning; b) Score range 0-100, higher scores represent more severe symptoms. | | | | | | |

| **Supplementary file 4.** Mean [standard deviations] health-related quality of life (HRQOL)-scores of 193 patients with a follow-up of >12 months who underwent gastrectomy for cancer compared to the general Dutch population. | | | | | | |
| --- | --- | --- | --- | --- | --- | --- |
|  | **Total**  ***n* = 193** | | **Reference population**  ***n*= 1731** | | **WMD** | ***p*-value** |
| **Quality of life questionnaire (QLQ)-C30** |  |  |  |  |  |  |
|  |  |  |  |  |  |  |
| **Global quality of life^a^** | 73 | [21] | 78 | [17] | -5 | **0.003** |
|  |  |  |  |  |  |  |
| **Functional scales^a^** |  |  |  |  |  |  |
| Physical | 78 | [20] | 90 | [15] | -12 | **<0.001** |
| Role | 72 | [30] | 89 | [21] | -17 | **<0.001** |
| Emotional | 80 | [24] | 89 | [16] | -9 | **<0.001** |
| Cognitive | 80 | [22] | 92 | [15] | -12 | **<0.001** |
| Social | 80 | [27] | 94 | [16] | -14 | **<0.001** |
|  |  |  |  |  |  |  |
| **General symptom scales^b^** |  |  |  |  |  |  |
| Fatigue | 35 | [27] | 17 | [20] | +18 | **<0.001** |
| Nausea and vomiting | 14 | [22] | 2.7 | [10] | +11 | **<0.001** |
| Pain | 20 | [27] | 15 | [22] | +5 | 0.020 |
| Dyspnoea | 19 | [26] | 7.1 | [17] | +12 | **<0.001** |
| Insomnia | 22 | [30] | 14 | [23] | +8 | **<0.001** |
| Appetite loss | 22 | [33] | 3.3 | [12] | +19 | **<0.001** |
| Constipation | 11 | [22] | 4.8 | [14] | +6 | **<0.001** |
| Diarrhoea | 18 | [27] | 3.9 | [14] | +14 | **<0.001** |
| Financial difficulties | 16 | [28] | 3.1 | [13] | +13 | **<0.001** |
|  |  |  |  |  |  |  |
| Scores are presented as mean [±SD]. a) Score range 0-100, higher scores represent a better quality of life or level of functioning; b) Score range 0-100, higher scores represent more severe symptoms. | | | | | | |
